# Prevalence and genomic characterization of *Salmonella* isolates from commercial chicken eggs retailed in traditional markets in Ghana

**DOI:** 10.3389/fmicb.2023.1283835

**Published:** 2023-11-01

**Authors:** Edward W. Archer, Tom Chisnall, Kwaku Tano-Debrah, Roderick M. Card, Samuel Duodu, Angela Parry-Hanson Kunadu

**Affiliations:** ^1^Nutrition and Food Science Department, University of Ghana, Accra, Ghana; ^2^Food and Drug Authority, Food Safety Management Department, Accra, Ghana; ^3^Animal and Plant Health Agency, Addlestone, United Kingdom; ^4^Biochemistry Cell and Molecular Biology Department, University of Ghana, Accra, Ghana; ^5^West African Centre for Cell Biology of Infectious Pathogens (WACCBIP), University of Ghana, Accra, Ghana

**Keywords:** *Salmonella*, eggs, Ghana, AMR, prevalence, serovar

## Abstract

*Salmonella enterica* are important foodborne bacterial pathogens globally associated with poultry. Exposure to *Salmonella*-contaminated eggs and egg-related products is a major risk for human salmonellosis. Presently, there is a huge data gap regarding the prevalence and circulating serovars of *Salmonella* in chicken eggs sold in Ghana. In this study, 2,304 eggs (pools of six per sample unit) collected from informal markets in Accra, Kumasi and Tamale, representing the three ecological belts across Ghana, were tested for *Salmonella*. Antimicrobial susceptibility testing and Whole Genome Sequencing (WGS) of the isolates were performed using standard microdilution protocols and the Illumina NextSeq platform, respectively. The total prevalence of *Salmonella* was 5.5% with a higher rate of contamination in eggshell (4.9%) over egg content (1.8%). The serovars identified were *S.* Ajiobo (*n* = 1), *S.* Chester (*n* = 6), *S.* Hader (*n* = 7), *S. enteritidis* (*n* = 2); and *S.* I 4:b:- (*n* = 8). WGS analysis revealed varied sequence types (STs) that were serovar specific. The *S.* I 4:b:- isolates had a novel ST (ST8938), suggesting a local origin. The two *S. enteritidis* isolates belonged to ST11 and were identified with an invasive lineage of a global epidemic clade. All isolates were susceptible to ampicillin, azithromycin, cefotaxime, ceftazidime, gentamicin, meropenem, and tigecycline. The phenotypic resistance profiles to seven antimicrobials: chloramphenicol (13%), ciprofloxacin (94%), and nalidixic acid (94%), colistin (13%), trimethoprim (50%) sulfamethoxazole (50%) and tetracycline (50%) corresponded with the presence of antimicrobial resistance (AMR) determinants including quinolones (*gyrA* (D87N), *qnrB*81), aminoglycosides (*aadA1*), (*aph(3“)-Ib aph(6)-Id*), tetracyclines (tet(A)), phenicols (*catA1*), trimethoprim (*dfrA14 and dfrA1*). The *S. enteritidis* and *S.* Chester isolates were multidrug resistant (MDR). Several virulence factors were identified, notably cytolethal distending toxin (*cdtB* gene), *rck*, *pef* and *spv* that may promote host invasion and disease progression in humans. The findings from this study indicate the presence of multidrug resistant and virulent strains of *Salmonella* serovars in Ghanaian chicken eggs, with the potential to cause human infections. This is a critical baseline information that could be used for *Salmonella* risk assessment in the egg food chain to mitigate potential future outbreaks.

## Introduction

*Salmonella* is a Gram negative, rod-shaped, and non-spore forming bacterium with facultative respiratory metabolism and made up of two species, *Salmonella bongori* and *Salmonella enterica* (Tindall et al., 2005; Su and Chui, 2007). Among the six subspecies of *Salmonella enterica*, subspecies *enterica* is responsible for 99% of salmonellosi*s* in humans and warm-blooded animals (Issenhuth-Jeanjean et al., 2014; Lamas et al., 2018). The non-typhoidal *Salmonella* (NTS) serovars include all serotypes except those that cause typhoid fever, such as *Salmonella Typhi* and *Salmonella Paratyphi* (Feasey et al., 2012). Globally, NTS pathogens are significant foodborne hazards affecting about 94 million people with an average incidence rate of 1.14% episodes per person (Majowicz et al., 2010; Murray et al., 2012). Although mainly associated with self-limiting enteric gastroenteritis, some NTS can become systemic and invasive, causing sepsis and other life- threatening bloodstream infections, especially in malnourished children, HIV-AIDS, and severe malaria patients, and may require antimicrobial treatment (MacLennan and Levine, 2013; Kariuki et al., 2015).

Poultry and poultry products are important carriers of NTS pathogens and potentially responsible for most foodborne zoonotic *Salmonella* transmission. Consumption of raw or undercooked eggs or food items containing raw eggs are a leading cause of many non-typhoidal salmonellosis outbreaks worldwide (European food safety authority and European Centre for Disease Prevention and Control (EFSA & ECDC), 2014). *S. enteritidis* is the serovar responsible for many egg-related *Salmonella* infections in humans (Hofer, 2021), and has a greater potency to contaminate the egg content through vertical transmission (Martelli and Davies, 2012). Since the mid-1980s, *S. enteritidis* has dominated most public health discussions in Europe, US, and many other developed countries (Gantois et al., 2008; Wales and Davies, 2011). It was the most prevalent serovar in many surveys carried out on *Salmonella* contamination of table eggs from 1991 to 2010 across different countries (Martelli and Davies, 2012). The wide spread of this pathogen is speculated to be related to the import of poultry breeding stocks (Li et al., 2021). Nonetheless, both *S. typhimurium* and *S. enteritidis* colonize reproductive organs of hens and they are among the leading causes of Salmonellosis globally, but *S. typhimurium* it is more broadly associated with food from various sources including eggs (Whiley and Ross, 2015). In addition to *S. enteritidis* and *S. typhimurium*, there are several other non-typhoidal *Salmonella* serovars, that might contaminate eggs (Bäumler et al., 2000; Threlfall et al., 2014).

In Ghana, *Salmonella* serovars have been reported in food sources including beef, raw cow milk, meat, poultry and lettuce (Addo et al., 2011; Andoh et al., 2016; Parry-Hanson Kunadu et al., 2020; Quansah et al., 2020). Additionally, a high prevalence of *Salmonella* serovars (44%) was found in chicken layers and farm samples (Andoh et al., 2016). Most people in Ghana, like other places in Africa, depend on open traditional retail markets for their egg supply where handling and storage conditions may not be ideal. Improper hygiene practices are major underlying predisposing risk factors to egg-borne salmonellosis (Gormley et al., 2011; King et al., 2011). Notwithstanding, there is a significant knowledge gap about the incidence of *Salmonella* in chicken eggs in Ghana. Also, currently no national systems are in place to support *Salmonella* surveillance programs. Clearly, *Salmonella* in poultry, particularly in chicken eggs has received little attention in Ghana, partly because there is limited data on foodborne disease outbreaks associated with this pathogen. Although some studies suggest that NTS infections is a health challenge in Ghana, with serovars Enteritidis and Typhimurium isolated from patient samples (Andoh Ahmed et al., 2017; Dekker et al., 2018), there is an evidence gap about how *Salmonella* in eggs contributes to human infections in Ghana.

In this study, we investigated for the first time the serovar prevalence of *Salmonella* isolates from table eggs sold in retail markets across three ecological belts in Ghana. Whole genome sequencing and antimicrobial resistance testing were performed to identify the circulating genotypes and assess the potential relationships among the resistance phenotypes and presence of antimicrobial resistant determinants, accompanying genetic markers of bacterial virulence. The study was expected to help in establishing a baseline data, which is the first step for developing an effective strategy for the control of salmonellosis through the egg value chain.

## Materials and methods

### Study design and sampling

A cross-sectional study was conducted within a period of 11 months (October 2019–August 2020) in Accra, Kumasi, and Tamale representing the coastal, middle, and northern belts of Ghana, respectively. A total of 2,304 eggs were purchased from retailers in 30 traditional markets in the three study locations. From each retailer, six eggs were randomly selected without consideration to the source of eggs and placed into sterile stomacher bags. They were transported on ice to the Microbiology laboratory, Nutrition and Food Science Department, the University of Ghana for processing and analysis. From each pool of six eggs, both the eggshell and egg contents were analysed separately within 24 h after purchase. A sample size of 384 pools (six eggs per pool) was computed, using the equation outlined by Cochran (1963), with a 95% confidence interval and 0.05 absolute precision by assuming a hypothesized proportion of egg retailers as 0.5. Out of the 384 samples collected, 156 eggs were sampled each from Kumasi and Accra, and 72 from Tamale. In Tamale, there were fewer egg retailers present in the markets compared to Accra and Kumasi. The sampling was proportionally done based on number of retailers in each market. The proportion of positive samples and the 95% confidence interval of the proportions were calculated with GraphPad using the modified Wald method for confidence intervals.^1^

### Preparation of eggshell and egg content

The crush method was used to process the sample as previously described (Musgrove et al., 2005; Sodagari et al., 2019). Briefly, 70% ethanol was used to sanitize the blunt or wide end (air sac) region of each of the six eggs, which were air-dried, and aseptically crack-opened using a sterile blade at the sanitized region, ensuring that shell pieces did not contaminate the contents. The contents of the six eggs were transferred and pooled in a sterile stomacher bag. The insides of the shells were rinsed using sterile phosphate-buffered saline (PBS) to remove most of the adhering albumen that may exhibit significant antimicrobial activity (Musgrove et al., 2005). Shells of the six eggs from each sample unit were pooled into a sterile stomacher bag and crushed by hand massaging, then the bag with crushed eggshells was placed inside two additional stomacher bags to prevent leakage during further homogenization processing.

### Isolation and identification of *Salmonella* sp.

Isolation of *Salmonella* from the eggshell and contents was performed following the culture-based international reference standard method ISO 6579-1:2017 (Sodagari et al., 2019). Twenty-five grams (25 g) of each pool of crushed eggshell was added to 225 mL Buffered Peptone Water (BPW) (Oxoid, England) and homogenized in a stomacher for 1 min. The six pooled egg contents of the same sample unit were blended in a stomacher for 2 min, and subsequently, 25 mL of this mixture was homogenized for 1 min with 225 mL of BPW. The two homogenates were then incubated at 37°C for 48 h. Following the pre-enrichment, 1 mL of each homogenate was inoculated into 10 mL Mueller Kauffman Tetrathionate Broth (MKTTn broth) (Oxoid, England) and incubated at 37°C for 24 h. In the same way, 0.1 mL from each broth were inoculated into 10 mL Rappaport Vassiliadis broth (RV broth) and incubated at 41.5°C for 24 h. The enriched cultures in RVB and TTB were streaked onto Hektoen agar and Xylose Lysine Deoxycholate (XLD) agar and plates incubated at 37°C for 24 h. Plates were examined for presumptive *Salmonella* colonies which appeared on XLD as opaque/yellow, pink, or red colonies with or without black centres, while on HE plates as blue-green to blue colonies with black centres. Plates with no *Salmonella* colonies were re-incubated and examined over a 24 h period. Presumptive *Salmonella* were purified on nutrient agar and species identification confirmed by matrix-assisted laser desorption/ionization-time-of-flight mass spectrometry (MALDI-TOF-MS). The confirmed strains were cryopreserved using overnight growth cultures in LB broth supplemented with 25% glycerol.

### Antimicrobial susceptibility testing

Antimicrobial susceptibility testing was performed by broth microdilution using commercial plates (Sensititre EU Surveillance *Salmonella*/*Escherichia coli* EUVSEC plate, Thermo Fisher Scientific, Basingstoke, UK), according to the manufacturer’s instructions. Briefly, a suspension of each isolate was prepared to a density of 0.5 McFarland in 5 mL sterile deionized water and 10 μL was transferred to 11 mL of Mueller Hinton broth to obtain a target inoculum density of between 1 × 10^5^ and 1 × 10^6^ CFU/mL. Fifty microlitres were dispensed into each well of the microtitre plate and incubated at 35–37°C for 18 to 24 h. Fourteen antimicrobials were tested in this manner (sulfamethoxazole, trimethoprim, ciprofloxacin, tetracycline, tigecycline, azithromycin, nalidixic acid, ampicillin, cefotaxime, ceftazidime, meropenem, chloramphenicol, colistin, and gentamicin) and the MICs were recorded as the lowest concentration that prevented visible growth. *E. coli* NCTC 12241 (ATCC 25922) was used as control strain. MICs were interpreted using Epidemiological Cut-Off (ECOFF) values (EUCAST, 2021) as wild type or non-wild type (Schwarz et al., 2010). The wildtype refers to isolates that are not intrinsically resistant nor carry antimicrobial resistance genes or mutations and are fully susceptible; whiles the non-wildtype refer to isolates that carry mutations or acquired resistance genes, which exhibit reduced susceptibility to a drug or antimicrobial.

Breakpoints proposed by the European Food Safety Authority (EFSA) (2014) were employed for azithromycin, colistin, and tigecycline as ECOFF values were not available. Isolates were defined as multidrug resistant when non-wild type for three or more classes of antimicrobial was observed (Schwarz et al., 2010), although it is acknowledged that non-wild type resistance does not necessarily correspond with clinical resistance.

### Whole genome sequencing and analysis

DNA extracts were prepared from overnight Luria broth (LB) cultures using either the commercial MagMAX^™^ CORE extraction kit (Thermo Fisher Scientific, Basingstoke, UK) with the semi-automated KingFisher Flex system (Thermo Fisher Scientific, Basingstoke, UK) or the biolate method (Wimalarathna et al., 2013). Extracted DNA was processed for whole genome sequencing (WGS) at APHA Central Sequencing Unit (APHA Weybridge, UK). Libraries were prepared with a Nextera XT DNA sample preparation kit (Illumina, San Diego, CA), according to the manufacturer’s instructions. WGS was carried out using the Illumina NextSeq platform (Illumina Inc., San Diego, California, United States) for short read sequencing. The resulting raw sequences were analyzed using the Nullabor 2 pipeline (Seemann, 2014), to produce *de novo* read assemblies and genome annotation. *S. enterica* serotype Enteritidis strain Durban (accession number CP007507) was used as reference. The presence of genes and point mutations conferring AMR were assessed using APHA SeqFinder (Anjum et al., 2016) and AMRFinderPlus (Feldgarden et al., 2019). SeqSero2 (Zhang et al., 2019) was used to determine the serovar of *Salmonella* isolates from the WGS. The Sequence Type (ST) was determined with MLST (version 2.19.0; https://github.com/tseemann/mlst) using the pubMLST database (Jolley et al., 2018). Virulence gene presence was assessed using Abricate^2^ and the virulence factor database (Chen et al., 2016) in Nullarbor.

The relatedness of isolates from four of the serovars detected (*S.* Chester, *S.* Hadar, *S.* Enteriditis, and *S.* I 4, 12:b, see results) was assessed using Snippy (version 4.6.0) and SNPdist (version 0.8.2). For each serovar the analysis included published genomes of the corresponding serovar from West Africa and a serovar-specific reference strain. The *S.* Chester reference genome was strain ATCC11997 (accession number CP019178.1), alongside ten published *S.* Chester genomes (Supplementary Table S1). The reference genome for *S.* Hadar was strain FDAARGOS_313 (accession number GCA_002209205.2), with 30 published *S.* Hadar genomes (Supplementary Table S2). *S. enteritidis* strain Durban (accession number CP007507) was used as reference and 27 published *S. enteritidis* genomes were included in the analysis (Supplementary Table S3). The *S.* I 4, 12:b isolates were compared to each other only, using isolate G05 as reference (Supplementary Table S4).

The whole genome sequences were deposited in the NCBI SRA under BioProject accession number PRJNA978427.

## Results

### Prevalence of *Salmonella* in retailed table eggs

In this study, 26 *Salmonella* isolates were identified from the 384 eggshell samples and 384 egg contents, representing an overall prevalence of 5.47% (Table 1). *Salmonella* was detected in both the eggshell (*n* = 19; prevalence of 4.95%) and egg contents (*n* = 7; prevalence of 1.82%). Five of the samples were positive for *Salmonella* spp. in both the eggshell and content. The prevalence of *Salmonella* was similar for Accra and Kumasi, whereas Tamale recorded lower positivity with no detection from egg content (Table 1). Little variation in prevalence by sampling period was observed, and the highest was in the months of January to March.

**Table 1:** Prevalence of *Salmonella* in table eggs at retail.

Variable	Categories	No. of samples tested	No. of positive samples	Prevalence of *Salmonella* (%)	95% Confidence interval
	**Eggshell + Egg content**	384	21	5.47	3.56 to 8.26
**Total**	Eggshell	384	19	4.95	3.15 to 7.64
	Egg content	384	7	1.82	0.81 to 3.79
Accra	Egg shell	156	9	5.77	2.92 to 10.74
	Egg content	156	4	2.56	0.78 to 6.63
Kumasi	Egg shell	156	8	5.13	2.47 to 9.95
	Egg content	156	3	1.92	0.04 to 5.76
Tamale	Egg shell	72	2	2.78	0.19 to 10.15
	Egg content	72	0	0	0.00 to 6.07
Sampling period	January–March	46	4	8.70	2.90 to 20.86
	April–June	90	3	3.33	0.73 to 9.75
	July–September	114	6	5.26	2.20 to 11.24
	October–December	134	8	5.97	2.88 to 11.51

### Diversity of *Salmonella* from table eggs in Ghana

To further characterise the isolates, they were initially examined by traditional serotyping methods to determine the serovar., however as testing proceeded, ten isolates returned a partial formula of 4,12:b and were *d*-tartrate positive (data not shown). This is consistent with *S. paratyphi* B var. Java, a causative agent for paratyphoid fever. Consequently, and with regard to containment requirements, serotyping was completed for all isolates using WGS methods, and MIC testing carried out only for the 16 non-*S.* 4,12:b isolates.

Twenty-four of the 26 isolates were examined by WGS and serovars established by SeqSero as: *S.* Ajiobo (*n* = 1), *S.* Chester (*n* = 6), *S.* Hader (*n* = 7), *S. enteritidis* (*n* = 2); and the isolates with a partial formula of 4,12:b were identified as *S.* I 4:b:- (*n* = 8). The *S.* Chester, *S.* Hader, *S. enteritidis*, and *S.* I 4:b:- isolates were present in eggshells and contents, whereas the *S.* Ajiobo isolate was recovered only from an eggshell sample (Table 2). Sequence Type (ST) correlated with serovar (Table 3; Supplementary Table S5): *S.* Ajiobo (ST951), *S.* Chester (ST1954), *S.* Hader (ST33), and *S. enteritidis* (ST11). The *S.* I 4:b:- isolates had a new ST, comprised of known alleles: *aroC*(179), *dnaN*(165), *hemD*(8), *hisD*(195), *purE*(140), *sucA*(207), and *thrA*(22) and was assigned as ST8938 by the Enterobase database (Zhou et al., 2020).

**Table 2:** Serovars of *Salmonella* obtained from eggshells and eggs content.

*Salmonella* serovars (sequence type)	Sample type	
	Egg shell	Egg content
Ajiobo (ST951)	1	0
Chester (ST1954)	5	1
Enteritidis (ST11)	1	1
Hadar (ST33)	4	3
I 4:b:- * (ST8938)	6	4

* The S. I 4:b:- serovar for one isolate from eggshell and one isolate from egg content was determined by traditional serotyping only.

**Table 3:** Correspondence between antimicrobial resistances determined by MIC (mg/L) and the presence of AMR genetic determinants.

Isolate ID	Serotype	Sequence Type	Chloramphenicol		Quinolones			Sulfamethoxazole		Tetracycline		Trimethoprim	
			MIC	Genotype	CIP MIC	NAL MIC	Genotype	MIC	Genotype	MIC	Genotype	MIC	Genotype
G17	Ajiobo	951	8		0.03	4		16		2		0.25	
G01	Chester	1954	8		**0.25**	**16**	*qnrB19*	**1,024**	***sul2***	**64**	*tet*(A)	**32**	***dfrA14***
G03	Chester	1954	8		**0.25**	**16**	*qnrB19*	**1,024**	***sul2***	**64**	*tet*(A)	**32**	***dfrA14***
G07	Chester	1954	8		**0.5**	**16**	*qnrB19*	**1,024**	***sul2***	**64**	*tet*(A)	**32**	***dfrA14***
G09	Chester	1954	8		**0.5**	**16**	*qnrB19*	**1,024**	***sul2***	**64**	*tet*(A)	**32**	***dfrA14***
G10	Chester	1954	8		**0.25**	**>128**	*qnrB19*	**1,024**	***sul2***	**64**	*tet*(A)	**32**	***dfrA14***
G26	Chester	1954	8		**0.5**	**16**	*qnrB19*	**1,024**	***sul2***	**64**	*tet*(A)	**32**	***dfrA14***
G16	Enteritidis	11	**128**	***catA1***	**0.25**	**>128**	*gyrA* (D87N)	**1,024**	***sul1*; *sul2***	**64**	*tet*(A)	**32**	***dfrA1***
G19	Enteritidis	11	**128**	***catA1***	**0.25**	**>128**	*gyrA* (D87N)	**1,024**	***sul1*; *sul2***	**64**	*tet*(A)	**32**	***dfrA1***
G02	Hadar	33	8		**0.25**	**>128**	*gyrA* (S83Y)	8		**64**	*tet*(A)	0.25	
G04	Hadar	33	8		**0.5**	**>128**	*gyrA* (S83Y)	8		**64**	*tet*(A)	0.25	
G08	Hadar	33	8		**0.25**	**>128**	*gyrA* (S83Y)	8		**64**	*tet*(A)	0.25	
G11	Hadar	33	8		**0.25**	**>128**	*gyrA* (S83Y)	8		**64**	*tet*(A)	0.5	
G13	Hadar	33	8		**0.25**	**>128**	*gyrA* (S83Y)	8		**64**	*tet*(A)	0.25	
G14	Hadar	33	8		**0.25**	**>128**	*gyrA* (S83Y)	8		**64**	*tet*(A)	0.25	
G15	Hadar	33	8		**0.25**	**>128**	*gyrA* (S83Y)	8		**64**	*tet*(A)	0.25	

Non-wildtype MIC values (mg/L) are indicated in bold and associated resistance genes detected shown; a blank cell indicates no corresponding gene detected. CIP, ciprofloxacin; NAL, nalidixic acid.

The three most observed serovars were *S.* Chester, *S.* Hadar, and *S.* I 4:b:- and to examine the diversity of each in more detail we assessed their relatedness through core genome SNP analysis. Five of the six *S.* Chester isolates were closely related with ≤12 SNPs difference and several isolates had ≤4 SNPs difference (Supplementary Table S1), and therefore met proposed relatedness threshold criteria (Schürch et al., 2018), indicating that these isolates are likely to be representatives of a single clone. The *S.* Chester isolate G10 was more distant (119–122 SNPs), but nevertheless was more closely related than ten published *S.* Chester genomes from West African countries (Benin, Gambia, and Nigeria) which had 6,725–13,869 SNPs difference (Supplementary Table S1). Similarly, the seven *S.* Hadar isolates are likely to be representatives of a single clone as they had 0–6 SNPs difference; and were most closely related publicly available *S.* Hadar genomes from West Africa were from Senegal with 91–149 SNPs difference (Supplementary Table S2). The *S.* I 4:b:- isolates possessed greater diversity between each other with 14–166 SNPs difference and did not meet proposed relatedness criteria to indicate they were representatives of a single clone (Supplementary Table S4).

**Figure 1:**
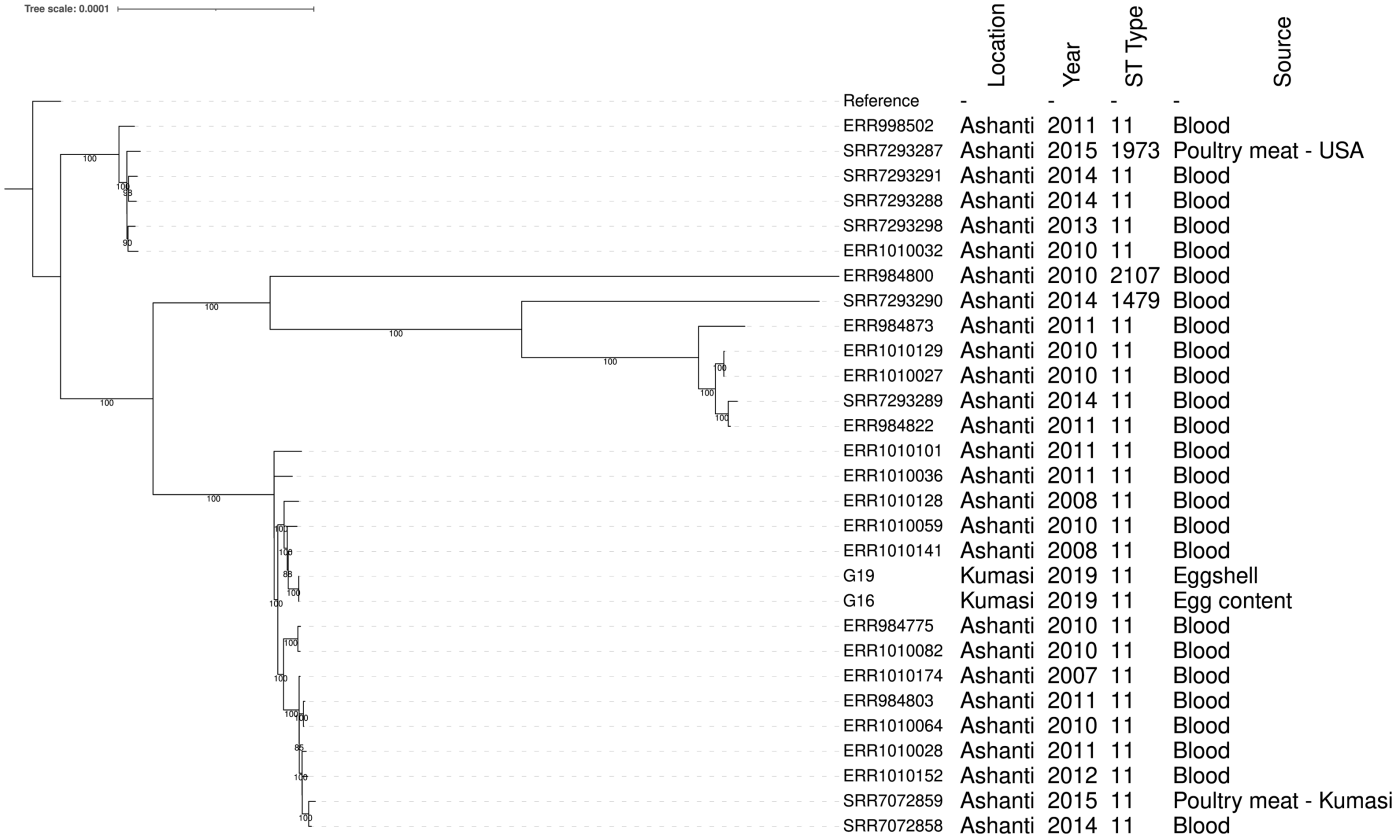
Phylogenetic tree showing the relationship between *S. enteritidis* isolates from eggs and patient clinical samples in the Ashanti region, Ghana. The *S. enteritidis* strain Durban (accession number CP007507) was used as the reference strain. Tree branch bootstrap values greater than 80 are shown. The MLST details and the sources of the isolates including the year of isolation are indicated.

The two *S. enteritidis* genomes were compared to *S. enteritidis* genomes isolated from clinical samples in Ghana and two poultry isolates from Ghana (Aldrich et al., 2019) in a core genome phylogenetic tree (Figure 1). The two *S. enteritidis* isolates from this study are likely to be representative of a single clone as they had 0 SNP difference (Figure 1; Supplementary Table S3). They were also sampled on the same day from a market in Kumasi, so it is possible that the same clone contaminated the pooled eggs. The isolates were most closely related to a Ghanian *S. enteritidis* ST11 isolate from a human blood infection obtained in 2008 (Figure 1; Supplementary Table S3), and which has been identified with the invasive lineage of a global epidemic clade and is fluoroquinolone resistant (Aldrich et al., 2019; Park et al., 2021).

### Antimicrobial susceptibility and carriage of resistance genes

The sixteen *Salmonella* isolates from serovars Ajiobo, Chester, Enteritidis, and Hadar were tested for antimicrobial susceptibility against a panel of 14 antimicrobials. All isolates were susceptible to ampicillin, azithromycin, cefotaxime, ceftazidime, gentamicin, meropenem, and tigecycline (Supplementary Table S5). The *S.* Ajiobo isolate was susceptible to all 14 antimicrobials, whereas the seven *S*. Hadar isolates were resistant to ciprofloxacin, nalidixic acid, and tetracycline (Table 3; Supplementary Table S5). The *S. enteritidis* and *S.* Chester isolates had resistance to three or more classes of antimicrobial and were consequently classified as multidrug resistant (MDR): *S. enteritidis* (chloramphenicol, colistin, ciprofloxacin, nalidixic acid, sulfamethoxazole, trimethoprim, and tetracycline); *S.* Chester (ciprofloxacin, nalidixic acid, sulfamethoxazole, trimethoprim, and tetracycline) (Table 3; Supplementary Table S5).

There was high correspondence between phenotypic antimicrobial resistance and the presence of AMR determinants (Table 3). For quinolone antibiotics, high nalidixic acid MIC values (>128 mg/L) were associated with the presence of point mutations in the quinolone-resistance-determining regions (QRDRs) of DNA gyrase (*gyrA*) resulting in changes to the amino acid sequence: *gyrA* (D87N) in *S. enteritidis* and *gyrA* (S83Y) in *S*. Hadar. The lower nalidixic acid MIC values (16 mg/L) in *S.* Chester were associated with the presence of *qnrB19* (Table 3). *S.* Chester isolate G10 had a nalidixic acid MIC of >128 mg/L, but harboured only *qnrB19* and no point mutations in the QRDRs. Tetracycline resistance was associated with *tet*(A), trimethoprim resistance with *dfrA1* (*S. enteritidis*) or *dfrA14* (*S.* Chester), and sulfamethoxazole resistance with *sul2* (*S.* Chester) or *sul1* and *sul2* (*S. enteritidis*). The two *S. enteritidis* isolates carried *catA1* and were resistant to chloramphenicol. Additional resistance genes conferring resistance to antimicrobials not tested by MIC in this study were present in many isolates (Supplementary Table S5). All isolates had *aac6-Iy*, a chromosomal-encoded aminoglycoside acetyltransferase in *S. enterica*, which requires a regulatory mutation to confer resistance (Magnet et al., 1999). The aminoglycoside resistance genes *aph(3″)-Ib* and *aph(6)-Id* were present in *S.* Chester, *S. enteritidis*, and *S.* Hadar (Supplementary Table S5). Additionally, the *S. enteritidis* isolates harboured the aminoglycoside resistance gene *aadA1*. The *S*. Ajiobo and *S*. I 4:b:- isolates carried the fosfomycin resistance gene *fosA7.2* (Supplementary Table S5).

### Virulence factors analysis

**Figure 2:**
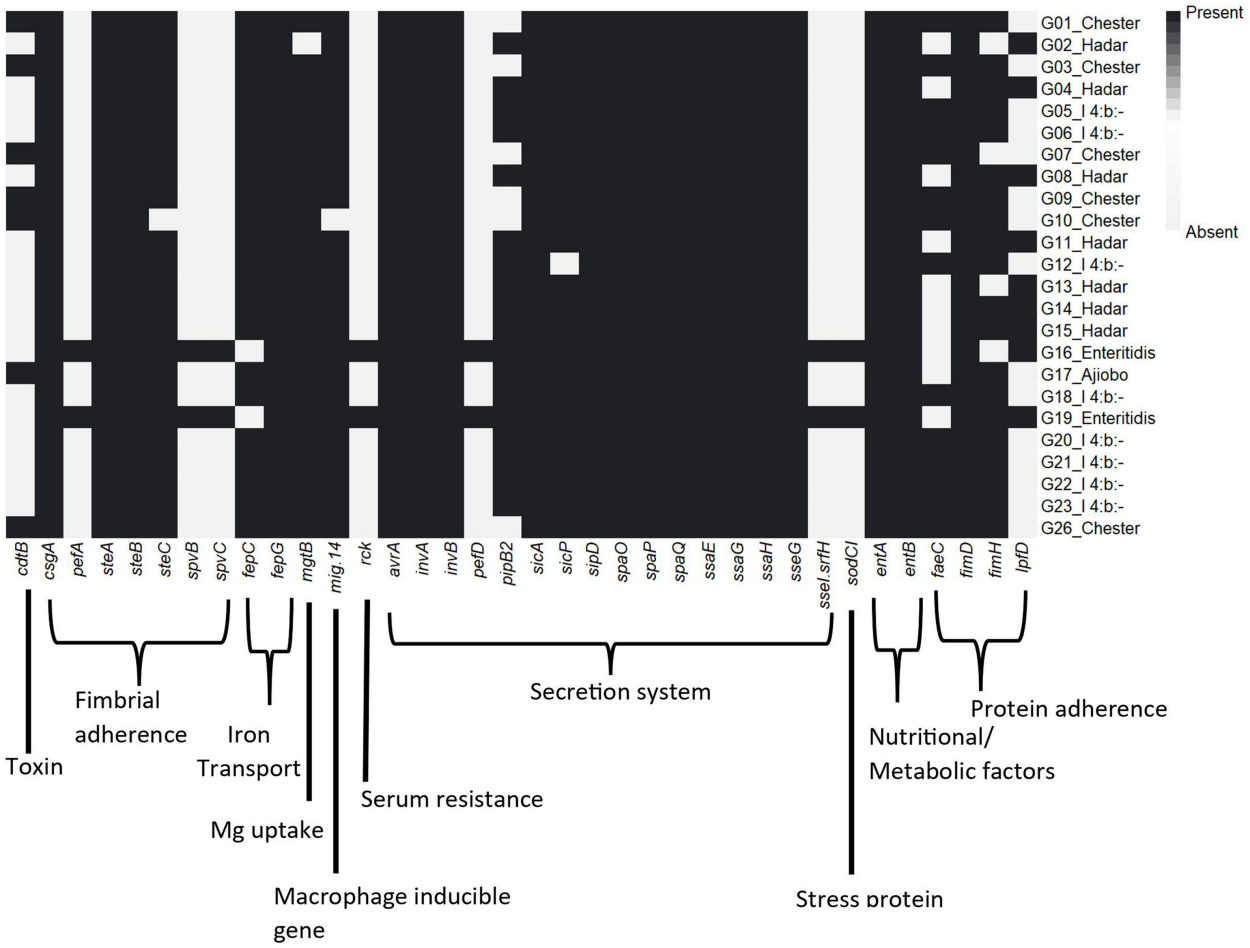
Heatmap of virulence genes distribution amongst the *Salmonella enterica* isolates.

Several virulence genes related to *Salmonella* pathogenicity were identified among the different serovars (Supplementary Table S6). A total of 66 virulence genes were well conserved across all serovars, which included genes encoding for host cell adhesion (e.g., *csg* and *fim* genes), cell invasion (e.g., *omp*, *org*, and *sip* genes), invasins (e.g., *inv* genes), effectors (e.g., *avrA*), and secretion systems (e.g., *prgJ*) (Figure 2). The *S.* Enteriditis isolates additionally carried *pefABCD* (fimbrial adherence determinants), ssel/srfH (type III secretion)*, sodCI* (stress survival), spvBCR (encoding secretory effector proteins/exotoxin) and *rck* (serum resistance). The cytolethal distending toxin B gene *cdtB* was present in the *S.* Chester and *S.* Ajiobo isolates.

## Discussion

This study investigated the prevalence, serovars, AMR patterns, virulence and genomic diversity of *Salmonella* isolated from table eggs sold in retail markets across three regions in Ghana. It has widely been reported that the prevalence of *Salmonella* in commercial table eggs is lower in developed countries (Carrique-Mas et al., 2008). For example, as low as 0.005% *Salmonella* prevalence was reported in table eggs in the US (Ebel and Schlosser, 2000), 0.47% in Europe (EFSA/ECDC, 2019), 0.3% in Japan (Sasaki et al., 2011), 0.04% in Northern Ireland (Murchie et al., 2007), 1% in Spain (Messens et al., 2006), and 1.8% in New Zealand (Wilson, 2007). These rates of detection are far lower than the overall *Salmonella* prevalence of 5.47% observed in the present study. On the contrary, the prevalence rates reported from nations in Asia and Africa are relatively higher including 7.7% in India (Suresh et al., 2006), 7.4% in South Korea (Lee et al., 2013), 7.0% in South Africa (Jambalang et al., 2017), 20.4%–24.2% in Nigeria (Ifeanyichukwu et al., 2016; Tessema et al., 2017), 10.5% in Ethiopia (Assefa et al., 2011), 13.3% in Bangladesh (Mahmud et al., 2016), and 15.0% in Sri Lanka (Kalupahana et al., 2017).

Personal observations in Ghana indicate that maintaining a cold chain storage facility for eggs during transport from the farm gate and at the retail level is a challenge. Moreover, eggs are kept for longer periods in open markets, thereby increasing the chance of microbial contamination due to unhygienic handling practices, favourable temperatures and humidity conditions for bacterial multiplication.

The detection of *Salmonella* in eggs may be influenced by the pre-analysis procedures adopted. Previous studies have reported on the use of different pre-enrichment media for the detection of *S. enteritidis* in shell eggs (Zhang et al., 2013a,b). Pre-enrichment in tryptic soy broth (TSB) was found to significantly improve culture sensitivity for Salmonella detection compared to other standard media such as nutrient broth and BPW. Although this seems promising as alternative protocol, they were based on experimentally contaminated samples. There are concerns regarding the use of artificial inoculated samples in any comparison or validation of microbial detection and enumeration methods, especially where other background microorganisms occur which may influence detection sensitivity. In this study, the ISO method 6,579–1:2017 with BPW pre-enrichment was adopted (Sodagari et al., 2019). This is a globally accepted standard analytical method that allows for Salmonella detection and isolation from both naturally and artificially contaminated food and environmental samples (Hara-Kudo et al., 2001; Mooijman et al., 2019). Moreover, BPW is less nutritionally rich compared with TSB, thus more appropriate for our experimental design since low microbial loads were expected and we wanted to limit background microorganisms such as *Proteus* that can reduce *Salmonella* detection sensitivity.

We observed that *Salmonella* recovery from eggshell was higher than egg content, consistent with previous findings (Bajaj et al., 2003; Suresh et al., 2006; Kalupahana et al., 2017). Several local practices along the value chain from farmgate to market may facilitate S*almonella* transmission. For instance, the continuous re-use of paper crates, excessive handling including repackaging of eggs into smaller units at the marketplace and, display of eggs under direct sunlight during retail can greatly increase the chance of *Salmonella* contamination. Furthermore, the common use of razor blades and metallic sponge as cleaning tools to remove dried faecal matter on eggshells could lead to redistribution of *Salmonella* on the egg surface and the opening of pores on the eggshell for *Salmonella* invasion. Vertical or transovarian transmission of *Salmonella* is also made possible either during egg formation or post lay, with *Salmonella* penetrating the eggshell surface to reach the egg content (Messens et al., 2005; Gantois et al., 2008). Additionally, the rate of *Salmonella* infection in flocks will contribute to the presence of *Salmonella* in eggs, although data on prevalence is limited in Ghana. In one study of 75 layer farms, an overall flock prevalence of 44.0% was reported (Andoh et al., 2016), and in another study of 38 farms the farm prevalence was 13.2% and all positive samples were from layers (Abilla et al., 2021).

Although we could not clearly assign spatial effect on the distribution of the *Salmonella* isolates, it was observed that 50% were obtained in eggs sampled from Accra whereas 42 and 8% were from Kumasi and Tamale, respectively. Except in Tamale where *Salmonella* was isolated from the eggshell only, samples from Accra and Kumasi showed positive results for both eggshell and contents. The high rate of detection of *Salmonella* in egg samples from the Accra market could be due to eggs produced and stored relatively longer before reaching the market. Accra is a major hub for the egg business due to large demands hence major production sites, including Kumasi, transport their eggs to Accra for sale. The highest rates of *Salmonella* contamination were observed in October – December and January – March (Table 1). This coincided with Christmas and New year festivities (December – January), where there is high demand for table eggs within the country with high economic returns, attracting many people to engage in retail activities even with little or no basic food hygiene knowledge. Seasonal variations in *Salmonella* incidence in table eggs have been reported in India (Suresh et al., 2006).

This is the first time *S.* Chester, *S*. Hadar and *S.* I 4:b serovars are reported in table eggs in Ghana. Both Chester and Hadar have been associated with disease outbreaks in other countries. In Canada, *S*. Chester was responsible for an outbreak due to consumption of cheese, meat and gelatin derived from the head of a pig seasoned with spices (Taylor et al., 2012). It was also implicated in a multi-serovar *Salmonella* outbreak involving egg sandwiches in China (Guo et al., 2015). More recently, *S*. Hadar was the cause of an outbreak involving 33 people in the US after consuming ground turkey (CDC, 2021). Though *S*. Ajiobo is clinically associated with tubo-ovarian abscess and cysts formation in humans (Manning and Saridogan, 2009; Himeno et al., 2013), the single isolate of *S*. Ajiobo detected in the eggshell may be an environmental contaminant as it has been previously isolated from water in Ghana (Dekker et al., 2018) and also in cane rats in Nigeria (Oboegbulem and Okoronkwo, 1990).

The SNP analysis of the isolates suggests that the Chester and Hadar serovars may each be clonal. The recovery of these isolates from different time points and locations (Supplementary Tables S2, S3), indicates that they might have originated from single contamination source. In contrast, *S.* I 4:b isolates seem more genetically diverse possibly due to its broader host range including poultry, reptiles, fish, mushrooms and turtles (Aung et al., 2020). This serovar had a novel sequence type (ST8938) and shared some features with *d*-tartrate fermenting variant of *S. paratyphi* B, which can cause gastroenteritis in humans (Toboldt et al., 2013).

The two *S. enteritidis* isolates detected in this study had identical core SNP genomes, and were in the same clade as a published genomes of isolates collected from human patients residing within the same geographical area of the Ashanti region in 2008–2011 (Figure 1; Aldrich et al., 2019). The ability of *S. enteritidis* to reach the egg content indicates a potential risk of causing egg-borne salmonellosis, particularly in the population that may consume raw or undercooked eggs and egg products such as ice cream, mayonnaise and sunny-side-up fried eggs.

Reduced susceptibility to quinolone antibiotics was observed for all isolates except the single *S.* Ajiobo isolate; fluoroquinolone resistant *Salmonella* have been listed as a high priority pathogen by the World Health Organization (Tacconelli et al., 2018). Furthermore, reduced ciprofloxacin susceptibility has been reported for *Salmonella enterica* causing bloodstream infection in human patients in Ghana, including at a high incidence in *S. enteritidis* (Eibach et al., 2016). The *S. enteritidis* and *S.* Chester isolates were multidrug resistant, which has been associated with more serious disease in people (Parisi et al., 2018). Group D *Salmonella*, including *S. enteritidis*, have a degree of intrinsic resistance to colistin (European Food Safety Authority (EFSA) and European Centre for Disease Prevention and Control (ECDC), 2023), accounting for the reduced susceptibility observed in the two *S. enteritidis* from this study. Importantly, no resistance phenotype or genotype associated with ampicillin, azithromycin, cefotaxime, ceftazidime, gentamicin, meropenem, and tigecycline was detected. The fosfomycin resistance gene fosA7 has been reported as chromosomally located in *S.* Heidelberg, *S.* Derby, and *S.* Reading (Rehman et al., 2017; Wang et al., 2021). This was detected in *S.* Ajiobo and *S*. I 4:b:-, expanding the diversity of serovars in which this gene is known to reside. High antimicrobial use has been reported in poultry farms in Ghana (Andoh et al., 2016; Afakye et al., 2020), and it is possible that the resistances observed in this study reflect usage. However, this study was undertaken at retail and collection of on-farm antimicrobial usage data was beyond the study scope.

The importance of virulence genes in the pathogenicity of *Salmonella* is well recognised (Fàbrega and Vila, 2013). Most virulence genes identified in this study were associated with adherence and effector delivery/secretion systems and were present in most isolates. Of particular interest is the *cdtB* gene detected the *S.* Chester and *S.* Ajiobo isolates. The *cdtB* typhoid toxin causes DNA damage that results in cell cycle arrest, cellular distension, and apoptosis of a broad range of mammalian cell lineages (Lara-Tejero and Galán, 2000). Although previously reported in S. Chester (Ikhimiukor et al., 2022), *cdtB* has not been reported in *S.* Ajiobo.

In conclusion, this study has provided the first prevalence data for *Salmonella* in eggs at retail in Ghana and describe the serovars detected, filling an important public health data gap. We have provided evidence for multidrug resistance and virulence potentials of these isolates. Ongoing surveillance to assess *Salmonella* in eggs will help inform food safety and enable monitoring of the impact of interventions, such as reduction of antimicrobial use on farm. Additionally, the generation of similar data from human clinical isolates can help inform a One Health approach, to gauge the impact of egg-derived *Salmonella* on public health and could contribute to source tracing. The adoption of good hygienic practices such as regular cleaning and disinfection of eggs trays at the market could be an appropriate option to reduce the frequent occurrence of this pathogen in eggs. The risk to consumers can be further reduced by cooking correctly, refrigeration, avoiding cross-contamination and ensuring good personal hygiene.

## Data availability statement

The datasets presented in this study can be found in online repositories. The names of the repository/repositories and accession number(s) can be found in the article/Supplementary material.

## Author contributions

EA: Conceptualization, Investigation, Methodology, Writing – original draft. TC: Data curation, Formal analysis, Investigation, Methodology, Writing – original draft. KT-D: Conceptualization, Project administration, Supervision, Writing – review & editing. RC: Conceptualization, Data curation, Formal analysis, Investigation, Methodology, Resources, Supervision, Writing – original draft, Writing – review & editing. SD: Conceptualization, Data curation, Formal analysis, Investigation, Methodology, Resources, Supervision, Validation, Writing – original draft, Writing – review & editing. AK: Conceptualization, Investigation, Methodology, Resources, Supervision, Writing – review & editing.

## Footnotes

^1^ https://www.graphpad.com/quickcalcs/confInterval1/

^2^ https://github.com/tseemann/abricate
